# Supplementary material for: Local ecdysone synthesis in a wounded epithelium sustains developmental delay and promotes regeneration in Drosophila
Source: bioRxiv. 2024 Feb 26:2024.02.25.581888. Preprint. [Version 1] doi: 10.1101/2024.02.25.581888 (PMC10925115; doi:10.1101/2024.02.25.581888)
Supplement: Supplement 1 [file media-1.pdf]

Supplemental Figures and Legends

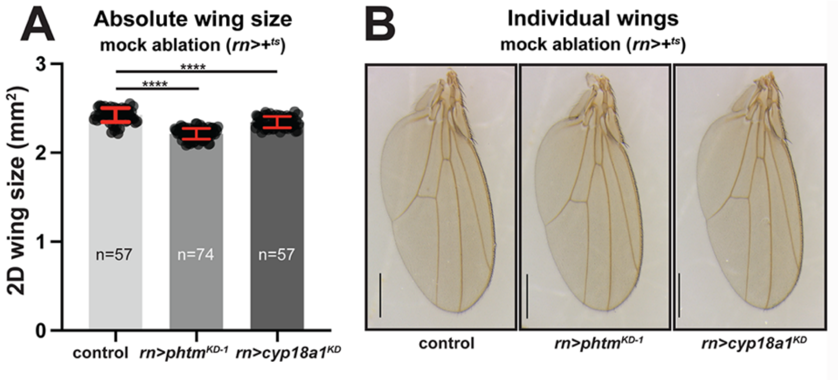

Figure S1. Local Ec knockdown in mock ablation.
(A) Absolute wing area data for mock ablated (*rn*>+) control, *pthm*$^{KD-1}$, and *Cyp18a1*^{KD}. Samples are female wings only. *n* values are cumulative from three separate crosses. **(B)** Representative mock ablated wings. Significance determined by Student's t-test. ****p<0.00001. Scale bars=0.5mm. Error bars=s.e.m.

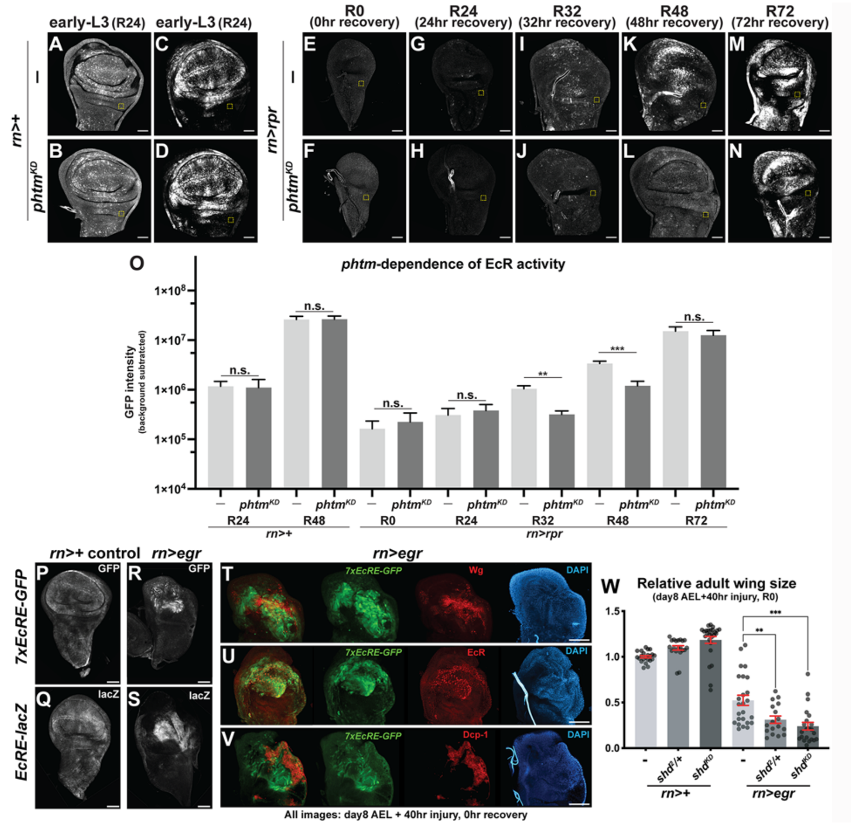

Figure S2. Levels of EcR activity across a time course of *rn>rpr* regeneration and *shade*-dependence of EcR activity induced by the *rn>egr*^{ts} system.

(**A-N**) Representative images of *7xEcRE-GFP* expression patterns in wing discs without (**A,C,E,G,I,K,M**) or with (**B,D,F,H,J,L,N**) *phtm*^{KD} at the indicated time points post-heat shock.
(O) Quantification of *7xEcRE-GFP* expression in mock injured *control* discs at the *phtm*-sensitive R24-R48 timepoints compared to regenerating (*rn>rpr*^{ts}) discs throughout a R0-R72 time course. **mock R24**: control n=14, *pthm*$^{KD-2}$ n=15; **mock R48**: control n=12, *pthm*$^{KD-2}$ n=12; **injured R0**: control n=9, *pthm*$^{KD-2}$ n=9; **injured R24**: control n=9, *pthm*$^{KD-2}$ n=9; **injured R32**: control n=21, *pthm*$^{KD-2}$ n=16; **injured R48**: control n=18, *pthm*$^{KD-2}$ n=20; **injured R72**: control n=19, *pthm*$^{KD-2}$ n=13. Data collected in three independent experiments. Y-axis in (**O**) is log(10) scale of fluorescence (arbitrary units). Significance determined by Student's t-test ***p*<.005, ****p*=0.0007, *n.s.*=not significant. Error bars=s.e.m. **(B-E)** Representative images of *7xEcRE-GFP* and *EcRE-lacZ* expression patterns in mock (*rn>+*^{ts}) and Eiger-injured (*rn>egr*^{ts}) discs. Scale bars=50µm. **(F-H)** *rn>egr*^{ts} induced expression of the *7xEcRE-GFP* reporter (green) imaged with antibodies to (**F**) Wg (red), (**G**) EcR (red; all isoforms), and (**H**) cleaved Dcp1 caspase. **(I)** Effect of *shd*^{2}/+ or *shd*^{KD} on wing size of *control* (*rn>egr*^{ts}) or Eiger-injured wings (*rn>egr*^{ts}). Sample sizes: injured (*rn>egr*^{ts}) n=25; *shd*^{2}/+ injured n=17; *shd*$^{KD-2}$ injured n=22. Sizes were wings normalized to mock ablated without *shd* allele. Samples are female wings only. Student's t-test. ***p*<0.001, ****p*<0.0001. Error bars=s.e.m. Scale bars=50µm. For *rn>egr*^{ts} wounding, larvae were temperature shifted at Day 8 for 40h and dissected for analysis at 0R.

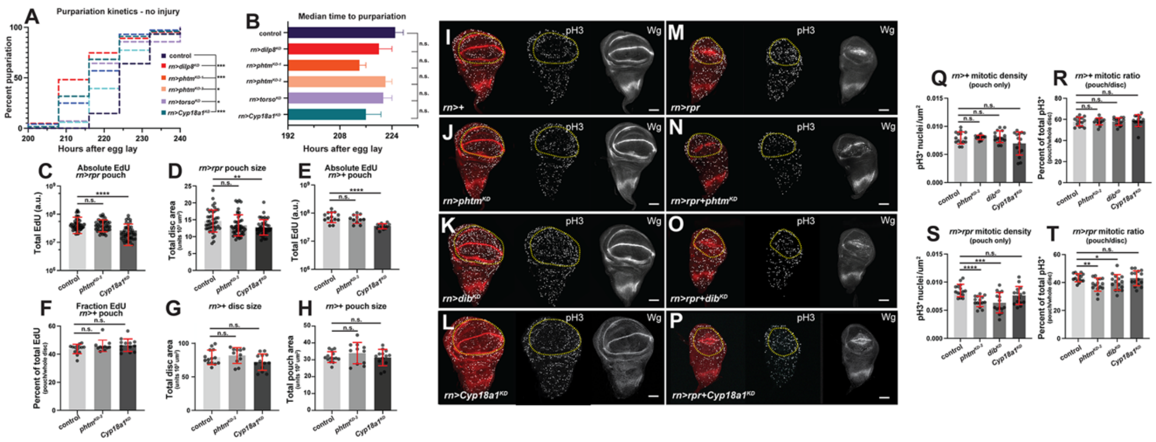

Figure S3. Pupariation timing of uninjured larvae and analysis of EdU and mitotic index following Rpr injury.

(A) Kaplan-Meyer survival curve for pupariation time-points following mock ablation (*rn*>+) with significance determined by the Log-rank (Mantel-Cox) test. Data are the sum of three independent experiments: *control* n=136, *pthm*$^{KD\text{-}1}$ n=158; *pthm*$^{KD\text{-}2}$ n=237; *Cyp18a1*^{KD} n=163; *torso*^{KD} n=98; *ilp8*^{KD} n=184. **(B)** Median time to pupariation following mock ablation (*rn*>+) of the genotypes in (**A**). One-way Anova with Dunnett's test used to determine statistical significance. ****p*<0.0001, **p*<0.05, *n.s.*=not significant. **(C)** Total internal EdU pouch fluorescence (a.u. log[10]) and (**D**) two-dimensional (2D) pouch size from injured (*rn>rpr*^{ts}) wing discs expressing *phtm*$^{KD\text{-}2}$ or *Cyp18a1*^{KD}. Data from three separate experiments: control n=45, *pthm*$^{KD\text{-}2}$ n=47, *Cyp18a1*^{KD} n=43. Student's t-test: *n.s.*=not significant. Error bars=s.d. Yellow dotted lines indicating the region of quantification based on anti-Wg staining (red) for this and following panels. (**E**) Total internal EdU fluorescence (a.u. log[10]) in mock injured (*rn*>+^{ts}) with the indicated RNAi lines. (**F**) Pouch fraction of EdU, (**G**) overall disc size, and (**H**) pouch size of mock *control* (*rn*>+^{ts}) mock ablated discs expressing *phtm*$^{KD\text{-}2}$ or *Cyp18a1*^{KD}. Data gathered in three separate experiments: control n=18, *pthm*$^{KD\text{-}2}$ n=16, *Cyp18a1*^{KD} n=16. Student's t-test: *****p*<0.0001, ***p*<.005, n.s.=not significant. Error bars=s.d. (**I-P**) Images of anti-pH3 staining (greyscale) in **(I-L)** control (*rn*>+^{ts}) discs or (**M-P**) *rn>rpr*^{ts} discs in combination with *pthm*$^{KD\text{-}2}$, *dib*^{KD}, or *Cyp18a1*^{KD}. Data were gathered in three independent experiments: control n=22, *pthm*$^{KD\text{-}2}$ n=27, *dib*^{KD} n=18, *Cyp18a1*^{KD} n=16. Student's t-test: n.s.=not significant. Error bars=s.d. **(M-P)** Representative images of pH3^{+} staining in control, *pthm*$^{KD\text{-}2}$, *dib*^{KD}, and *Cyp18a1*^{KD} ablated (*rn>rpr*^{ts}*)* wing discs. **(Q,S)** Mitotic density (pH3^{+} nuclei/pouch μm^{2}) and (**R,T**) mitotic ratio (pH3^{+} pouch/pH3^{+} total) of mock injured (*rn*>+^{ts}) or injured (*rn>rpr*^{ts}) discs with or without *phtm*$^{KD\text{-}2}$ or *Cyp18a1*^{KD}. Data gathered in three seperate experiments: *control*, n=41; *pthm*$^{KD\text{-}2}$, n=37; *dib*^{KD}, n=44; *Cyp18a1*^{KD}, n=24. Student's t-test: *****p*<0.0001, ****p*=0.0005, ***p*<.005, **p*<0.05, n.s.=not significant. Error bars=s.d. Scale bars=50μm.

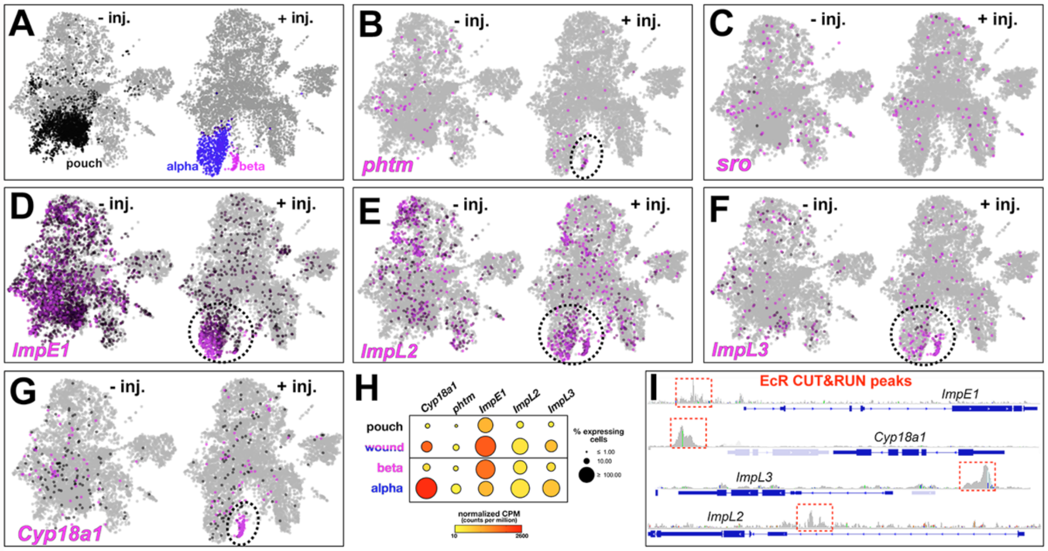

Figure S4. Ilp8-GFP levels in mock ablation and epithelial barrier permeability in *pthm*^{KD}. UMAP plots of larval wing discs generated from a public single cell sequencing database (https://scope.aertslab.org/#/WingAtlas/WingAtlas) with annotations for (**A**) pouch (black), and alpha wound (blue) or beta wound (magenta) (as defined in Floc'hlay et al., 2023). (**B-G**) UMAP plots of *phtm*, *sro*, *ImpE1*, *ImpL2* and *ImpL3* mRNA expression in uninjured (left) vs. injured (right) larval wing discs. Dotted circles highlight enrichment in alpha/beta wound populations. (**H**) Bubble plots representation of data in **B, D-G**. (**I**) IGV views of EcR Cut&Run peaks mapped using a public dataset (Uyehara and McKay, 2019).

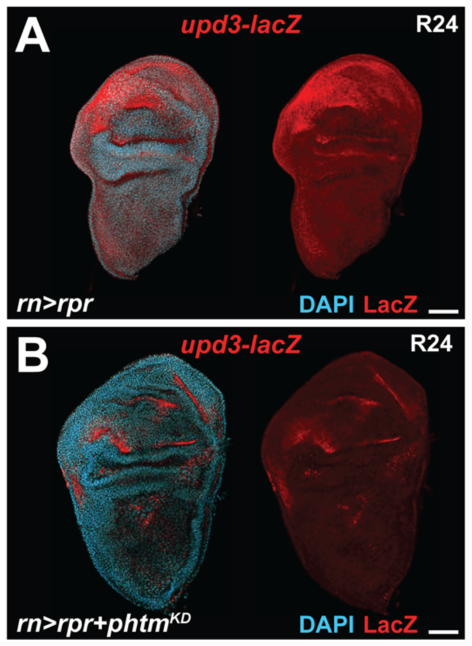

Figure S5. Optical projection of *phtm*^{KD} effect on *upd3-lacZ* expression in *rn>rpr*^{ts} injured discs. Z-stack projection of two R24 *rn>rpr*^{ts} injured discs carrying the *upd3-lacZ* reporter and co-stained with DAPI. (**A)** Injury alone, (**B**) with *phtm*^{KD} included (red=anti ßgal, cyan=DAPI).

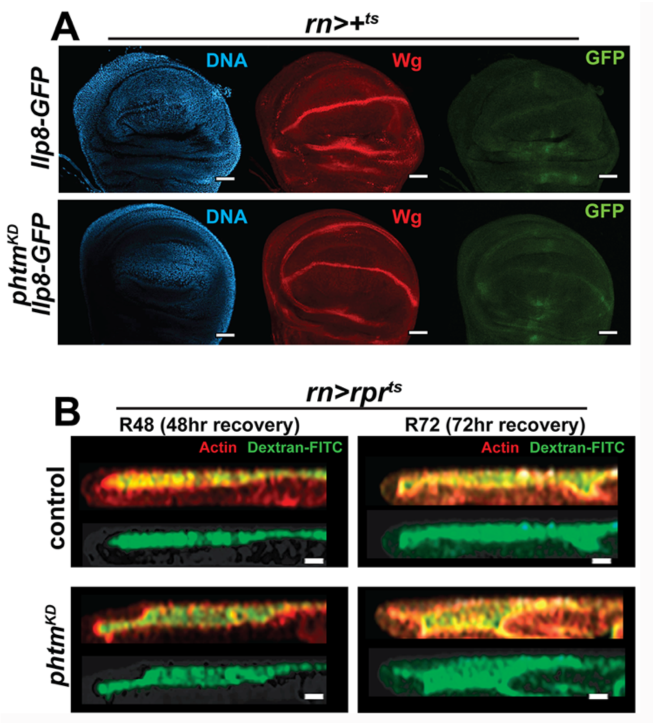

Figure S6. ***Ilp8-GFP*** **expression and epithelial permeability are unaffected by** ***pthm*^{KD}.**

(A) Representative images of *Ilp8-GFP* expression (green), DAPI (blue) and Wg (red) in mock injured (*rn*>+^{ts}) wing discs with and without *phtm*^{KD}. (**B**) Representative orthogonal views through the wing pouch of injured R48 or R72 discs +/- *phtm*^{KD} incubated with fluorescein (FITC) conjugated dextran (green) and co-stained with phalloidin (F-actin; red). Phalloidin staining marks actin of peripodial and disc proper cells, which enclose a central lumen. FITC-dextran accumulates to similar levels in the lumens of injured R48 and R72 discs with or without concurrent *phtm*^{KD}. Scale bars=10μm.

Table S1. Sequences for DNA primers for qPCR. Related to Figure 4. Pairs of DNA sequences (listed 5'-to-3') used to assay expression levels of the corresponding mRNAs

**REAGENT or RESOURCE**	**SOURCE**	**IDENTIFIER**
Primer pair for *rp49*:<br>GCTAAGCTGTCGCACAAATG<br>GTTCGATCCGTAACCGATGT	Integrated DNA Technologies	N/A
Primer pair for *dilp8*:<br>TGGTCATCGGAGTCTGTTGC<br>TTTTGCCGGATCCAAGTCGA	Integrated DNA Technologies	N/A
Primer pair for *ets21c*:<br>GTGCCAACAGAGGCCGATTA<br>CTGTTGGTGGGAACTTCCGT	Integrated DNA Technologies	N/A
Primer pair for *upd3*:<br>GCTGACCTTCCAGCAGAAAT<br>TGCTGTGCGTTTCGTTCA	Integrated DNA Technologies	N/A
